# Factors influencing the uptake of cardiac rehabilitation by cardiac patients with a comorbidity of stroke

**DOI:** 10.1016/j.ijcha.2020.100471

**Published:** 2020-02-06

**Authors:** A.S. Harrison, N.J. Gaskins, L.A. Connell, P. Doherty

**Affiliations:** a The University of York, United Kingdom; b Faculty of Health and Wellbeing, University of Central Lancashire, United Kingdom; c East Lancashire Hospitals NHS Trust, Burnley General Hospital, United Kingdom

**Keywords:** Rehabilitation, Stroke, Uptake/participation, Comorbidities

## 1 Introduction

Cardiac rehabilitation (CR) is an integral part of the treatment offered to people diagnosed with coronary heart disease (CHD). It is a complex secondary prevention intervention that aims to reduce risk factors, promote a healthy lifestyle and improve quality of life [1]. Patients become eligible for assessment for CR following diagnosis of a cardiac event, such as myocardial infarction (MI) or heart failure, or after coronary revascularization. The effectiveness of CR was summarised in a recent Cochrane review of trials which reported a reduction in CV mortality and readmissions [2] and Cardiac Rehabilitation Outcome Study (CROS) which used robust registry based observational studies concluding on CR benefiting overall mortality [3].

However, although CR is a well-established intervention, only 50% of the eligible population take up the offer to attend [4]. Previous research has investigated predictors of attendance for similar interventions in different populations and specific treatment groups such as Percutaneous Coronary Intervention (PCI) in CR [5].

In a study looking at the whole CR population, analysis split by gender identified that age, ethnicity and social deprivation were all significantly associated with starting CR [6]. Older patients, being of South Asian or other ethnicity, single and residing in a higher deprived area were all associated with a reduced likelihood of attendance.

In an aging population with an increasing prevalence of comorbidities, it is important to explore the predictors for attending CR in populations with particular comorbidities. CHD is categorised as a cardiovascular disease, as are peripheral arterial disease and stroke [7]. Stroke has been reported as a comorbidity in some of those people eligible for CR [4] and shares similar aetiology and modifiable risk factors with CHD [8], [9], [10].

People post-stroke often suffer from poor cardiovascular health and may have physical, cognitive and psychosocial impairments [11], [12]. There is emerging evidence that CR is both feasible and beneficial for people post-stroke [10], [13], [14], [15] although it is potentially underused for this population [14]. It is therefore important to identify factors that influence attendance at CR in this population. In the UK, 5.3% of the attending CR population have a comorbidity of stroke [4].

The aim of this study was to identify and evaluate factors contributing to the likelihood of a cardiac patient with comorbid stroke attending CR in the UK.

## 2 Methods

This observational study utilised data collected for the National Audit of Cardiac Rehabilitation (NACR). Patient and service-level characteristics were included in the analysis to identify significant associations of patients with comorbid stroke attending CR. Hierarchical logistic regression models were built to assess the extent of the associations.

### 2.1 Data

The study’s data is from a routinely collected audit of CR, the National Audit of Cardiac Rehabilitation (NACR). The NACR collects data from CR programmes across the UK and has a 74% coverage for electronic data entry [4]. The electronic data was acquired in a link-anonymised format from 229 programmes, which collected data on patient’s demographics, risk factors and baseline measures prior to starting CR. The data collection of patient information is covered by 251 exemption that is reviewed annually by NHS Digital. The rationale for data collection is to improve the quality of CR service delivery for public benefit. Patients were included if they had an initiating cardiac event, such as myocardial infarction, between 1st January 2013 and 30th Jan 2019.

The primary variable of interest is whether the patient started Core CR (Phase 3); defined as the point where patients are assessed, goals agreed and patients begin their formal structured CR programme.

### 2.2 Statistical analysis

The analyses were conducted in IBM statistical package SPSS V.25. (SPSS, Chicago, Illinois, USA)

Correlation and group comparisons utilised t-tests and Pearson correlation respectively. Subject to having sufficient data to fulfil statistical distribution assessments (N > 30) all potential covariates were investigated in the analysis. Backwards stepwise logistic regression models were built to investigate whether, accounting for covariates, the patient-level and service factors were associated with the patient attending CR.

Relevant important covariates were included in the analysis, where they were evidenced in the literature or significant in preliminary analysis. Age (years), gender (male/female) and marital status (single/partnered) have been shown to influence the outcomes following a variety of different rehabilitation interventions, including CR. Marital status was documented as ’single’ for patients who were single, widowed or separated, and ‘partnered’ for those married, partnered or in civil partnership. Length of hospital stay, source of referral and local index of multiple deprivation (IMD) were all included as previous studies showed an association with engagement and attending CR [5], [6]. The IMD was split into quintiles, and compared the highest deprived areas to the 2nd, 3rd, 4th and 5th deprived quintiles accordingly. Service-level covariates including the multidisciplinary team and staff hours were analysed. A multidisciplinary team (MDT) was defined as having three or more different staff disciplines which aligns with the BACPR [1] core components of delivering CR. Staff hours along with relative size of programme were also input into the model as continuous variables.

Patient’s comorbidity status was included in the univariate analysis and in the regression analysis. Comorbidities were grouped into similar conditions as shown in Table 3 (detailed list provided in Appendix 1).

Statistical level for significance was p < 0.05 and actual significant values were expressed as reported up to 0.001. Due to the large number of univariate analyses performed, the p values in the univariate analysis were adjusted using Bonferroni correction which accounted for the sum of analyses 13, this changed the threshold to 0.004. Data model checking was performed to ensure that the models were a good fit through assumptions associated with the regressions.

## 3 Results

**Fig. 1:**
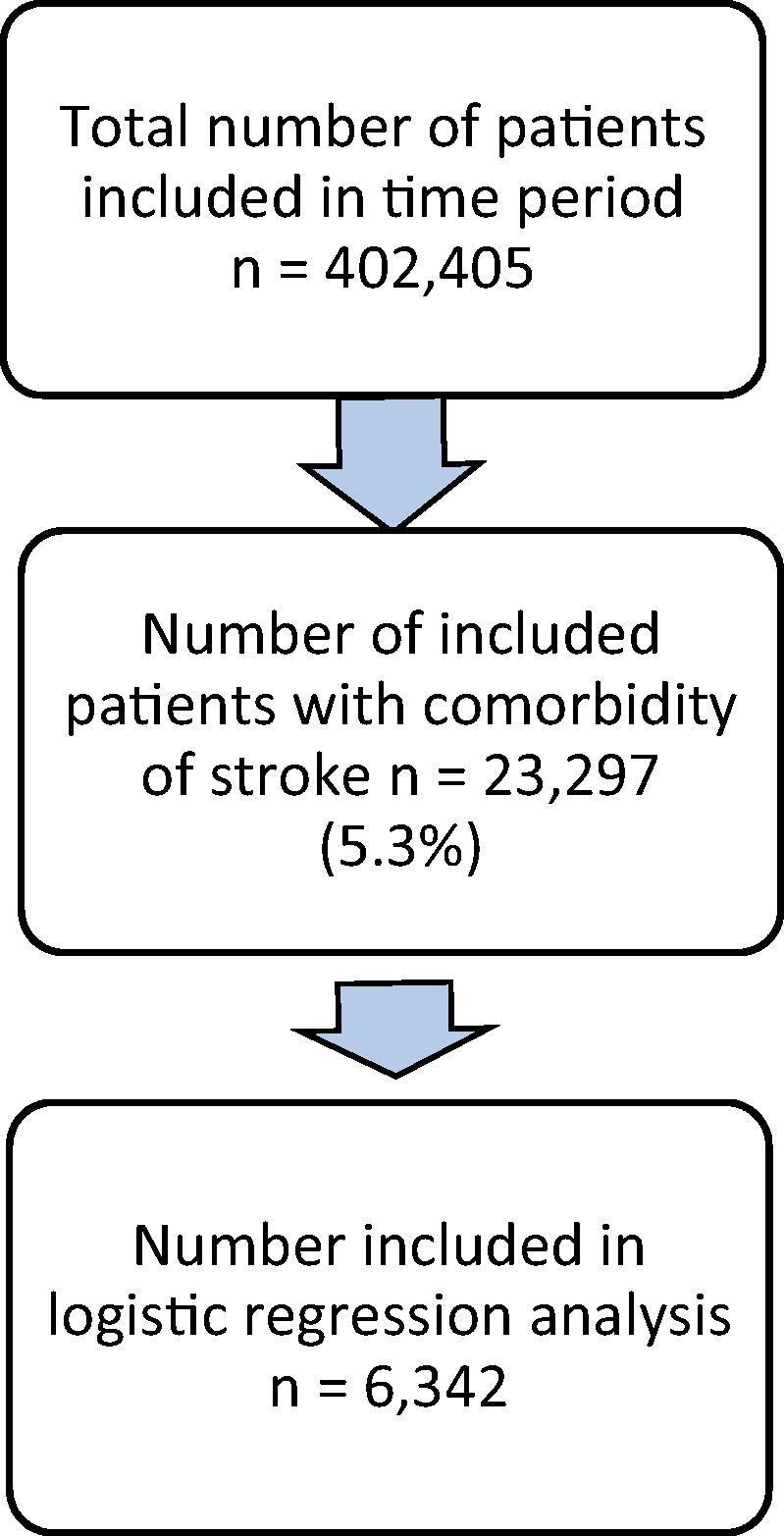
Flow diagram of the study population.

The total number of patients entered into the CR audit during the time period was 402,405, of which 23,297 (5.3%) had a comorbidity of stroke. Valid case selection for the regression resulted in 6,342 in the final regression model (Fig. 1).

Table 1 shows the descriptive of the population with comorbid stroke. This sub-population was divided into two groups: those who attended CR (44.46%) and those who did not (55.54%).

**Table 1:** 

		Did the Patient Start Rehab							
		Not Attending		Attending		Total			
		Mean/SD	Count	Mean/SD	Count	Mean/SD	Count	Mean Difference	P-value
Age Years		74 (11.39)	12,938	70 (10.55)	10,359	72 (11.19)	23,297	3.867	<0.001
Hospital Length of Stay days		11 (19.94)	9862	12 (26.99)	8371	12 (23.44)	18,233	0.601	0.084
Proportion of Stroke patients in Programme (%)		7.10 (2.47)	12,938	6.86 (2.56)	10,359	6.99 (5.51)	23,297	0.245	<0.001
Patients By Week		17.71 (11.85)	12,938	16.59 (11.26)	10,359	17.21 (11.60)	23,297	1.119	<0.001
Staff Hours Per Patient		12.44 (20.01)	9895	16.09 (26.49)	7641	14.03 (23.13)	17,536	3.648	<0.001
		Count	% Not Attending	Count	% Attending	Count	% by sub group	Chi-square	P-Value
Gender	Male	8198	53.00%	7280	47.00%	15,478	67.00%	124.382	<0.001
	Female	4620	60.70%	2988	39.30%	7608	33.00%		
Ethnicity	White	9497	54.10%	8047	45.90%	17,544	75.30%	56.598	<0.001
	Non-White	3441	59.80%	2312	40.20%	5753	24.70%		
Marital Status	Single	3097	59.40%	2120	40.60%	5217	31.40%	119.295	<0.001
	Partnered	5734	50.30%	5676	49.70%	11,410	68.60%		
Previous Cardiac Event	No	5851	54.80%	4818	45.20%	10,669	45.80%	3.838	0.05
	Yes	7087	56.10%	5541	43.90%	12,628	54.20%		
IMD quintiles	Lowest Quintile	2370	65.40%	1254	34.60%	3624	19.00%	172.181	<0.001
	Second Quintile	2226	59.50%	1514	40.50%	3740	19.60%		
	Third Quintile	2154	56.60%	1654	43.40%	3808	20.00%		
	Fourth Quintile	2113	54.70%	1747	45.30%	3860	20.30%		
	Fifth Quintile	2061	51.40%	1946	48.60%	4007	21.00%		
Multidisciplinary Team (MDT)	No MDT	2050	63.40%	1184	36.60%	3234	17.40%	115.825	<0.001
	MDT	8167	53.00%	7234	47.00%	15,401	82.60%		
Referring Staff	Hospital Based	9726	55.60%	7753	44.40%	17,479	88.20%	16.818	<0.001
	Primary Care Setting	1197	51.20%	1143	48.80%	2340	11.80%		
Recode Treatment as None, PCI, CABG, other	None	3444	72.20%	1329	27.80%	4773	20.49%	893.941	0.001
	PCI	4153	51.70%	3882	48.30%	8035	34.49%		
	CABG	812	36.90%	1388	63.10%	2200	9.44%		
	Other Treatment	4529	54.60%	3760	45.40%	8289	35.58%		

The average age was 72 years old, higher than in the wider CR population (67 years) and younger than the average age within the wider stroke population (77 years) [16]. Those who did not take up the offer of CR were on average 4 years older than those who did.

The gender split within the population was 67% male and 33% female, which is comparable with the wider CR population. However, this gender split is very different to the stroke population that had approximately 50/50 split across the time period. There were a greater number of females (60.7%) than males in the non-attender group in those with comorbid stroke. Similar to the full CR population, those in the stroke population of white ethnicity and having a partner dominated the total population (68.6%-84.5%). The non-attender group had a higher proportion of non-white and single (p =<0.001 and p=<0.001).

Deprivation, source of referral and any treatment during their admission influenced attendance at CR. High deprivation, referral from a hospital setting and lack of CVD treatment, e.g. PCI, CABG or other, were all negatively associated with attendance with a greater proportion in the non-attender group.

The programme specific data suggested that absence of an MDT, less staff hours per week and a greater number of total patients were associated with a reduction in uptake of CR. Attending a hospital where there was no MDT was linked with a 10% reduction in the number attending CR (p = <0.001).

Table 2 shows the comorbidity groups of those with comorbid stroke divided into attenders and non-attenders. The musculoskeletal, psychosocial problems and erectile dysfunction groups show a greater proportion of attenders, with the remaining groups showing reduced numbers.

**Table 2:** Comorbidities - grouped.

		Did the Patient Start Rehab					
		No		Yes		Total	
		Count	%	Count	%	Count	
Musculoskeletal Comorbidities	No	10,126	58.4%	7222	41.6%	17,348	74.5%
	Yes	2812	47.3%	3137	52.7%	5949	25.5%
Ischemia Comorbidities	No	9598	55.5%	7682	44.5%	17,280	74.2%
	Yes	3340	55.5%	2677	44.5%	6017	25.8%
Metabolic Comorbidities	No	7027	56.1%	5507	43.9%	12,534	53.8%
	Yes	5911	54.9%	4852	45.1%	10,763	46.2%
Cancer	No	11,762	55.7%	9343	44.3%	21,105	90.6%
	Yes	1176	53.6%	1016	46.4%	2192	9.4%
Hypertension	No	5841	55.8%	4634	44.2%	10,475	45.0%
	Yes	7097	55.4%	5725	44.6%	12,822	55.0%
COPD + Asthma	No	10,721	55.2%	8709	44.8%	19,430	83.4%
	Yes	2217	57.3%	1650	42.7%	3867	16.6%
Psychosocial Problems	No	12,205	57.0%	9193	43.0%	21,398	91.8%
	Yes	733	38.6%	1166	61.4%	1899	8.2%
Erectile Dysfunction	No	12,384	56.0%	9734	44.0%	22,118	94.9%
	Yes	554	47.0%	625	53.0%	1179	5.1%

The logistic regression model shown in Table 3 included 6,342 cases. The model identified 9 variables that were statistically significant associated with participation in CR. Patients’ age and ethnicity were negatively associated with participation with every year increase in age resulting in a 2.5% reduced likelihood of attendance. Those who were of non-white ethnicity had a 11.6% reduced likelihood (Age OR 0.975p = <0.001 and Ethnicity OR 0.884p = <0.001) of attendance.

**Table 3:** Logistic Regression results for attending core cardiac rehabilitation.

**Variables in the Equation**						
		B	Sig.	Exp(B)	95% C.I. for EXP(B)	
					Lower	Upper
Age at Initiating Event (years)		−0.025	0.000	0.975	0.970	0.980
Proportion of Stroke		−0.062	0.000	0.940	0.920	0.961
Ethnicity (White)		−0.123	0.096	0.884	0.765	1.022
Marital Status (Single)		0.177	0.003	1.194	1.064	1.340
Treatment (None)	PCI	0.771	0.000	2.162	1.826	2.560
	CABG	1.272	0.000	3.569	2.885	4.414
	Other	0.629	0.000	1.877	1.576	2.235
IMD (1st Quintile Most Deprived)	2nd Quintile	0.218	0.015	1.243	1.042	1.482
	3rd Quintile	0.424	0.000	1.527	1.281	1.822
	4th Quintile	0.581	0.000	1.788	1.502	2.128
	5th Quintile	0.658	0.000	1.930	1.620	2.301
Comorbidity Status (None)	Musculoskeletal	0.452	0.000	1.571	1.390	1.776
	Ischemic	−0.177	0.004	0.838	0.744	0.944
	Cancer	0.150	0.085	1.161	0.980	1.377
	COPD	−0.230	0.002	0.794	0.686	0.919
	Social Problems	0.357	0.001	1.430	1.163	1.757
	Erectile Dysfunction	0.254	0.043	1.289	1.008	1.649
Number of Patients seen per week		−0.014	0.000	0.986	0.982	0.990
Staff Hours per patient		0.019	0.000	1.019	1.012	1.026
Multidisciplinary team (No < 3)		0.494	0.000	1.638	1.406	1.908
Constant		0.496	0.037	1.642		

Hosmer Lemeshow sig. 0.865, Predictive Power 63.8%, Nagelkerke R Square 0.135.

If patients were partnered or had had a CHD treatment, there was a positive association with participation in CR. Having a partner increased likelihood of attendance by 19.4% (OR 1.194p = 0.003) and having any treatment had an 87.7% to 356.9% increased likelihood (Other OR 1.877P = <0.001 CABG OR 3.569P = <0.001).

The level of deprivation was significantly associated with patients’ participation, with reduction in deprivation incrementally increasing the likelihood. Moving from the most deprived to the second had a 24.3% increased likelihood, whereas for the most to the least deprived had a 93% increased likelihood (2nd OR 1.243p = 0.015 5th OR 1.930p = <0.001).

The comorbidity groups that were significant included musculoskeletal, ischaemic, cancer, COPD, social problems and erectile dysfunction.

The service-level factors, including proportion of stroke patients attending a programme, total number of patients at the site, MDT and total staff hours, were all statistically significant. The proportion of people with stroke and number of patients seen were negatively associated with 0.6% and 0.14% reduced likelihood respectively (p = <0.001). The MDT and total staff hours were positively associated with a 1.9% increased likelihood for every hour increase and 63.8% increase if a MDT was present (p =<0.001).

The model was a good fit with Hosmer Lemeshow p value = 0.865, the predictive power was 63.8%, the model assumptions were all met.

## 4 Discussion

This paper compared the sub-population of CR patients with comorbid stroke with the wider CR and stroke populations and identified and evaluated patient and service characteristics influencing likelihood of uptake of CR for these patients. This analysis included 23,297 patients with comorbid stroke, of which 6,342 cases were input into the regression model. The model identified that a mix of key patient and service-level factors were significantly associated with these patients attending CR.

The patient-level factors that were significant included age, ethnicity, marital status, treatment and local deprivation score. Presence of comorbidities such as musculoskeletal, cancer, psychosocial and erectile dysfunction in addition to stroke were associated with greater attendance, whereas having ischemic or COPD comorbidities were associated with reduced likelihood. CHD treatment, such as PCI or CABG, was linked with a 187.7–356.9% increased likelihood of attendance. Parallels exist with recent stroke research where those who had acute medical treatment (thrombolysis) received more intensive physiotherapy [17], although it is recognised that the provision of in-patient rehabilitation is different to the attendance at a CR programme. People from a less deprived area also had an increased likelihood.

This study’s results are similar to that of previous work in other CR populations, including PCI only patients, which suggested key patient predictors such as gender, age and ethnicity play an important role in patients taking part in CR [5], [6]. Previous research looking at the whole CR population or percutaneous coronary intervention (PCI) especially highlighted similar patient-level predictors to attend CR, however, this is the first time that these were found when programme variables were included. The severity of stroke can also impact on rehabilitation as those with mild or moderate stroke may receive more intensive physiotherapy, and therefore rehabilitation, than those with severe stroke [17].

In terms of service-level factors it was anticipated that a CR programmes’ staffing would be associated with the attendance rate. This study showed that, when factoring in the relative size (number of patients within the programme), for every hour increase of staff time there was a linked 1.9% increased likelihood of attendance by the patients. In addition to this, a programme with an MDT was associated with 63.8% greater likelihood of attendance. The unique findings of this study indicate that programmes with a more comprehensive and well-resourced MDT are more likely to succeed in increasing the attendance of patients with a comorbidity of stroke. In contrast with patient-level factors such as age and ethnicity, staffing is a modifiable factor. Investment in staffing may be a means of improving the attendance rate for people with comorbid stroke. What is not known from this study is whether poorer performing programmes have difficulties with staff recruitment in comparison with those which are performing well. Based on service-level criteria higher quality CR programmes have a greater uptake by people with comorbid stroke [18]. If staff recruitment is challenging then other models of delivery and service reorganisation need to be explored. This would require future qualitative research around different settings and intervention timings and content.

Stroke and CHD are intrinsically linked in their aetiology and modifiable risk factors. Research has shown that stroke patients benefit from adapted CR so the unequal provision of this intervention for people with comorbid stroke is not justifiable. The knowledge of modifiable service-level factors provided by this study is invaluable for future research investigating service reorganisation for the benefit of a diverse population such as this.

### 4.1 Limitations

Although this study was conducted well and accounted for multiple analysis, there were two limitations that have impacted the study.

The first of these are the level of missing data. Key variables such as area of residence and staffing profile including profession and level of expertise lead to a reduction in sample size when valid case analysis was used for the model. Even though the missing variables lead to a reduction in the sample, all cases were compared against the wider CR population and stroke population which allowed the authors to conclude that there was little chance of selection bias.

The second was the variable of comorbidity of stroke. This was a binary categorical variable which allowed the study to select the 5.3% of patients who had stroke in additional to their cardiovascular event. However, it would have been beneficial to also know the time since stroke and severity of the stroke amongst attenders and non-attenders. This information could be included in future research.

## 5 Conclusion

This study concluded that both patient- and service-level factors contributed to the likelihood of cardiac patients with comorbid stroke attending CR. The patient-level variables are consistent with wider CR literature on CR uptake, suggesting different models of delivery need to be explored to meet the diversity of the population. CR programmes with a more comprehensive and well-resourced MDT are more likely to succeed in increasing the attendance of patients with comorbidity of stroke. This highlights inequity of provision that is not justifiable. Strategies to overcome these modifiable factors should be explored.

## 6 Authorship statement

This author takes responsibility for all aspects of the reliability and freedom from bias of the data presented and their discussed interpretation.

## Funding

This study and the NACR data is funded by the British Heart Foundation grant (040/PSS/17/18/NACR). This work was also supported by a grant from the Chartered Society of Physiotherapy Charitable Trust (Grant No. PRF/17/B01).

## Declaration of Competing Interest

No conflicts of interest were present
